# Automated Radiology Report Labeling in Chest X-Ray Pathologies: Development and Evaluation of a Large Language Model Framework

**DOI:** 10.2196/68618

**Published:** 2025-03-28

**Authors:** Abdullah Abdullah, Seong Tae Kim

**Affiliations:** 1 Department of Computer Science and Engineering, Kyung Hee University, 1732 Deogyeong-daero, Giheung-gu, Yongin, Gyeonggi-do, 17104, Republic of Korea, 82 312013761

**Keywords:** large language model, generative pre-trained transformers, radiology report, labeling, BERT, thoracic pathologies, LLM, GPT

## Abstract

**Background:**

Labeling unstructured radiology reports is crucial for creating structured datasets that facilitate downstream tasks, such as training large-scale medical imaging models. Current approaches typically rely on Bidirectional Encoder Representations from Transformers (BERT)-based methods or manual expert annotations, which have limitations in terms of scalability and performance.

**Objective:**

This study aimed to evaluate the effectiveness of a generative pretrained transformer (GPT)-based large language model (LLM) in labeling radiology reports, comparing it with 2 existing methods, CheXbert and CheXpert, on a large chest X-ray dataset (MIMIC Chest X-ray [MIMIC-CXR]).

**Methods:**

In this study, we introduce an LLM-based approach fine-tuned on expert-labeled radiology reports. Our model’s performance was evaluated on 687 radiologist-labeled chest X-ray reports, comparing *F*1 scores across 14 thoracic pathologies. The performance of our LLM model was compared with the CheXbert and CheXpert models across positive, negative, and uncertainty extraction tasks. Paired *t* tests and Wilcoxon signed-rank tests were performed to evaluate the statistical significance of differences between model performances.

**Results:**

The GPT-based LLM model achieved an average *F*1 score of 0.9014 across all certainty levels, outperforming CheXpert (0.8864) and approaching CheXbert’s performance (0.9047). For positive and negative certainty levels, our model scored 0.8708, surpassing CheXpert (0.8525) and closely matching CheXbert (0.8733). Statistically, paired *t* tests indicated no significant difference between our model and CheXbert (*P*=.35) but a significant improvement over CheXpert (*P*=.01). Wilcoxon signed-rank tests corroborated these findings, showing no significant difference between our model and CheXbert (*P*=.14) but confirming a significant difference with CheXpert (*P*=.005). The LLM also demonstrated superior performance for pathologies with longer and more complex descriptions, leveraging its extended context length.

**Conclusions:**

The GPT-based LLM model demonstrates competitive performance compared with CheXbert and outperforms CheXpert in radiology report labeling. These findings suggest that LLMs are a promising alternative to traditional BERT-based architectures for this task, offering enhanced context understanding and eliminating the need for extensive feature engineering. Furthermore, with large context length LLM-based models are better suited for this task as compared with the small context length of BERT based models.

## Introduction

### Background

Radiology reports consist of expert observations of the radiologist based on the Chest X-ray images of the patient. These reports consist of free-text and unstructured information in the form of long paragraphs. The extraction of labels from unstructured radiology reports is the task of radiology report labeling and it provides us structured information which can be used for many downstream tasks such as medical report generation and natural language explanation generation. It also enables training of large-scale medical imaging models [1]. Previous works for labeling of radiology reports involve use of complicated feature engineering of medical domain knowledge [2]. and Bidirectional Encoder Representations from Transformers (BERT) based approaches [3]. Transformers have also demonstrated success in radiology report labeling [4,5]. However, all these methods have limitations which hinders their adoption in the clinical setting. In case of methods which use complex feature engineering, these methods have leveraged manual annotation to shift the burden from feature engineering, requiring considerable time and expertise. Furthermore, these methods do not take advantage of existing feature-engineered labelers, which are state-of-the-art on many medical tasks. On the other hand for methods using BERT based models, the models are limited by the inherent limitations of BERT models such as their noncausal nature and limited context length. BERT-based models, despite their effectiveness in text classification tasks, have two key architectural limitations that constrain their performance in radiology report labeling. First, BERT’s bidirectional nature focuses on context aggregation but lacks the ability to model causal relationships in sequential data. This noncausal nature can hinder its ability to fully capture the hierarchical and temporally dependent structure of radiology reports, where findings are often sequentially described. Second, BERT’s limited input context length (typically 512 tokens) prevents it from effectively processing the long and detailed narratives commonly found in radiology reports. As a result, crucial information in extended texts may be truncated, leading to incomplete or suboptimal labeling. These limitations reduce the adaptability of BERT-based methods to real-world radiology settings, where comprehensive understanding of the entire report is often required.

Large language models (LLMs) such as Qwen address these challenges by offering extended context lengths (several thousand tokens), allowing the model to process full radiology reports without truncation. In addition, their ability to incorporate causal reasoning and handle instruction-based tasks makes them particularly suitable for medical labeling tasks, where nuanced and ambiguous language is prevalent. Although BERT based methods have shown increased abilities in the classification and other natural language tasks, their architecture poses a hindrance to their use.

Smit et al [3] introduced a combination of existing radiology report labelers and expert annotations to achieve highly accurate automated radiology report labeling . Their approach consists of a bio-medically pretrained BERT model [6,7], which is trained on the outputs of an existing labeler. They call their resulting model CheXbert. While CheXbert has generated considerable results, it has been unable to capture the full diversity, complexity and the ambiguous nature of natural language in the radiology reports. Their BERT-based solution while providing remarkable performance on the task of labeling is limited in its context length and noncausal nature. Which means for longer radiology reports it fails to provide a solution.

In our work we propose an LLM based radiology report labeler. LLMs have proven to be successful in their natural language generation capabilities. These models also have longer context lengths which makes them highly suitable for natural language generation tasks. Furthermore, these LLMs are adept at following instructions and given proper instructions these LLMs can be made good labelers for the radiology reports. Our LLM-based model inherently provides ease of use in other LLM-based solutions for the medical domain enabling clinical automation.

Our generative pretrained transformer (GPT)-based LLM beats the BERT-based CheXbert model on many pathologies and with a far bigger context length can handle long reports as compared with CheXbert. Our model outperforms the previous labelers [8] for many pathologies on an external dataset, MIMIC-CXR [9]. Our method of training medical report labelers opens room for other labels and longer textual input which makes it broadly useful for natural language processing tasks within the medical domain.

### Related Work

Many natural language processing systems have been developed to extract structured labels from unstructured free-text radiology reports [2,10-16]. Mostly, these methods rely heavily on feature engineering and include strict vocabulary and grammatical rules to find and classify radiological reports. NegEx [17], a popular rule-based method, uses simple regular expressions for detecting negation of findings and is often used in combination with the Unified Medical Language System [18]. NegBio [19], an extension to NegEx, uses universal dependencies and subgraph matching for pattern definition and graph traversal search. It includes uncertainty detection in addition to negation detection for multiple pathologies in radiology reports, and is used to label the ChestX-Ray14 dataset [1]. The CheXpert labeler [8] improves upon NegBio on chest x-ray report classification by more controlled extraction and an improved Natural Language Processing framework and rules for uncertainty and negation extraction. The CheXpert labeler has been applied to generate labels for the CheXpert dataset and MIMIC-CXR [9], which are among the largest chest x-ray datasets publicly available. We use the MIMIC-CXR dataset to train our LLM-based framework and report our findings on a subset of the test set of MIMIC-CXR which has been labeled by expert radiologists.

Previous approaches have also been trained using radiology reports annotated by expert radiologists [20]. In these approaches, training data is limited by radiologist time and expertise. Chen et al [21] trained convolutional neural networks with Global Vectors for Word Representation [22] on 1000 radiologist-labeled reports for classification of pulmonary embolism in chest computed tomography scan reports and improved upon the previous rule-based peFinder [23,24]. trained both recurrent and convolutional networks in combination with attention mechanisms on 27,593 expert-annotated radiology reports. Transformer-based models have also been applied to the task of radiology report labeling [4], trained BERT [6] and XLNet-based [25] classifiers on 3856 radiologist labeled reports to detect normal and abnormal labels. Wood et al [5] proposed ALARM, an MRI head report classifier on head MRI data using BioBERT model [26] trained on 1500 radiologist-labeled reports. They demonstrate improvement over previous fixed embedding and word2vec-based [27] models [28] CheXbert labeler [3]. Also proposed a BERT based model which is trained on expert annotated radiology reports and achieves state of the art results for radiology report labeling. However, their method has limitations which include restriction to the context length of 512 which is the limitation of BERT based models.

Recent advancements in the application of LLMs across various domains, including medical informatics, have demonstrated their versatility and efficacy. Models such as GPT-3 and GPT-4 have been used for diverse tasks, including automated clinical note generation, question answering in health care, and medical coding, showcasing their ability to handle complex and domain-specific language tasks. In radiology, LLMs have been explored for summarizing imaging findings, generating patient-friendly explanations, and aiding in clinical decision-making, highlighting their potential beyond classification tasks. Furthermore, instruction-tuned LLMs, such as ChatGPT and specialized variants like BioGPT, have been shown to adapt effectively to biomedical domains, opening avenues for tasks such as multimodal data interpretation and real-time clinical assistance. These advancements emphasize the need for further exploration of LLMs’ contextual understanding and adaptability, particularly in radiology report labeling and other biomedical text processing tasks.

In our work we propose an LLM based solution to solve the task of biomedical text labeling. We not only propose an alternative to BERT based models which achieves better scores on certain labels and has far bigger context length than BERT based models but our approach can also be applied to other biomedical text labeling.

## Methods

### Task

Radiology report labeling is a critical task involving the extraction of information on the presence or absence of specific thoracic pathologies, such as consolidation or edema, from free-text radiology reports. This process enables the transformation of unstructured diagnostic text into structured data, facilitating clinical decision-making, research and the development of predictive models. In this task, a labeler processes the free-text radiology report as input and assigns 1 of 4 classes, blank, positive, negative, or uncertain, to each of 14 predefined observations, reflecting the certainty level for each prediction. A “positive” label indicates the presence of a pathology, while “negative” denotes its absence, and “uncertain” is used when the report is ambiguous about the condition. The “blank” class is assigned when no relevant information is available for an observation. By converting radiology reports into labeled data, this approach supports streamlined access to essential diagnostic insights and provides a valuable structured dataset that can be used to train and validate machine learning algorithms in medical imaging.

### Data

In radiology, there exist 2 large datasets of chest x-rays, CheXpert [8] (consisting of 224,316 images), and MIMIC-CXR [9] (consisting of 377,110 images). Both datasets have corresponding radiology reports that have been labeled for the same set of 14 observations using the CheXpert labeler [8] from the Impression section, or other parts of the radiology report. Furthermore, a subset of both datasets also contain manual annotations by expert radiologists. On CheXpert, a total of 1000 reports (CheXpert manual set) were reviewed by 2 board certified radiologists with disagreement resolution through consensus. On MIMIC-CXR, a total of 687 reports (MIMIC-CXR test set) were reviewed by 2 board certified radiologists and manually labeled for the same 14 medical pathology labels as in CheXpert. However, the radiology reports for the Chexpert dataset have not been made public. Due to nonavailability of radiology reports for the Chexpert dataset, we used the MIMIC-CXR test set for evaluation.

### Large Language Models

LLMs are built upon stacked decoder layers from the transformer architecture, often referred to as “auto-regressive models” because of their causal structure. This auto-regressive nature enables these models to predict each token sequentially, relying only on the preceding tokens as context. During training, LLMs learn the task of next-token prediction, where they must accurately anticipate the subsequent token in a sequence given the previous tokens as input. This prediction task is essentially a binary classification problem, where the model assesses whether its predicted token matches the correct ground truth token. The accuracy of each prediction is evaluated by calculating a cross-entropy loss, which measures the difference between the model’s output and the correct token. This loss is then back-propagated through the model to update its weights, refining its ability to generate contextually accurate and coherent responses over time.

LLMs are pretrained on extensive amounts of diverse text data from vast resources available on the internet. This extensive pretraining enables the models to capture complex patterns and detailed knowledge present in language, making them highly effective at understanding and generating natural language. Their exceptional performance in natural language generation, coupled with their ability to handle longer text inputs, motivates their application in various specialized domains, such as radiology report labeling.

In our work, we specifically use the Qwen model [29], particularly the Qwen1.5‐0.5B variant. This model demonstrates significant capabilities in various natural language generation tasks relative to its moderate size, making it both efficient and powerful. One of the Qwen model’s key advantages is its extensive context length of 32,000 tokens, meaning it can handle considerably larger text inputs as context compared with BERT-based models, which are typically limited to a 512-token context. This increased context capacity allows the Qwen model to process lengthy radiology reports or extended medical dialogues without truncating important information.

The enhanced context length in LLMs is particularly beneficial in clinical settings, where maintaining continuity in patient information, such as previous history or ongoing conversations, is essential. As LLM-based frameworks are increasingly adopted in health care, often in the form of biomedical chatbots and other automated systems, the capacity to retain extensive context is critical. Our model addresses this need, providing a solution that integrates seamlessly with existing LLM-driven tools in clinical environments. This ensures that radiology report labeling and other clinical tasks benefit from both accuracy and the ability to preserve a comprehensive, context-aware understanding of patient data.

### Instruction Fine-Tuning

To fully use the capabilities of our pretrained LLM, we fine-tune it using a specialized instruction dataset. This dataset is constructed from radiology reports paired with corresponding pathology labels, each with an associated certainty level from the MIMIC-CXR dataset. The goal of this instruction tuning is to guide the pretrained LLM to understand the relationships within radiology reports, enabling it to accurately identify pathologies and assign certainty levels. By providing targeted instructions, we aim to refine the LLM’s ability to interpret the clinical language and nuanced patterns within radiology reports, thereby enhancing its performance in radiology report labeling.

**Figure 1.**
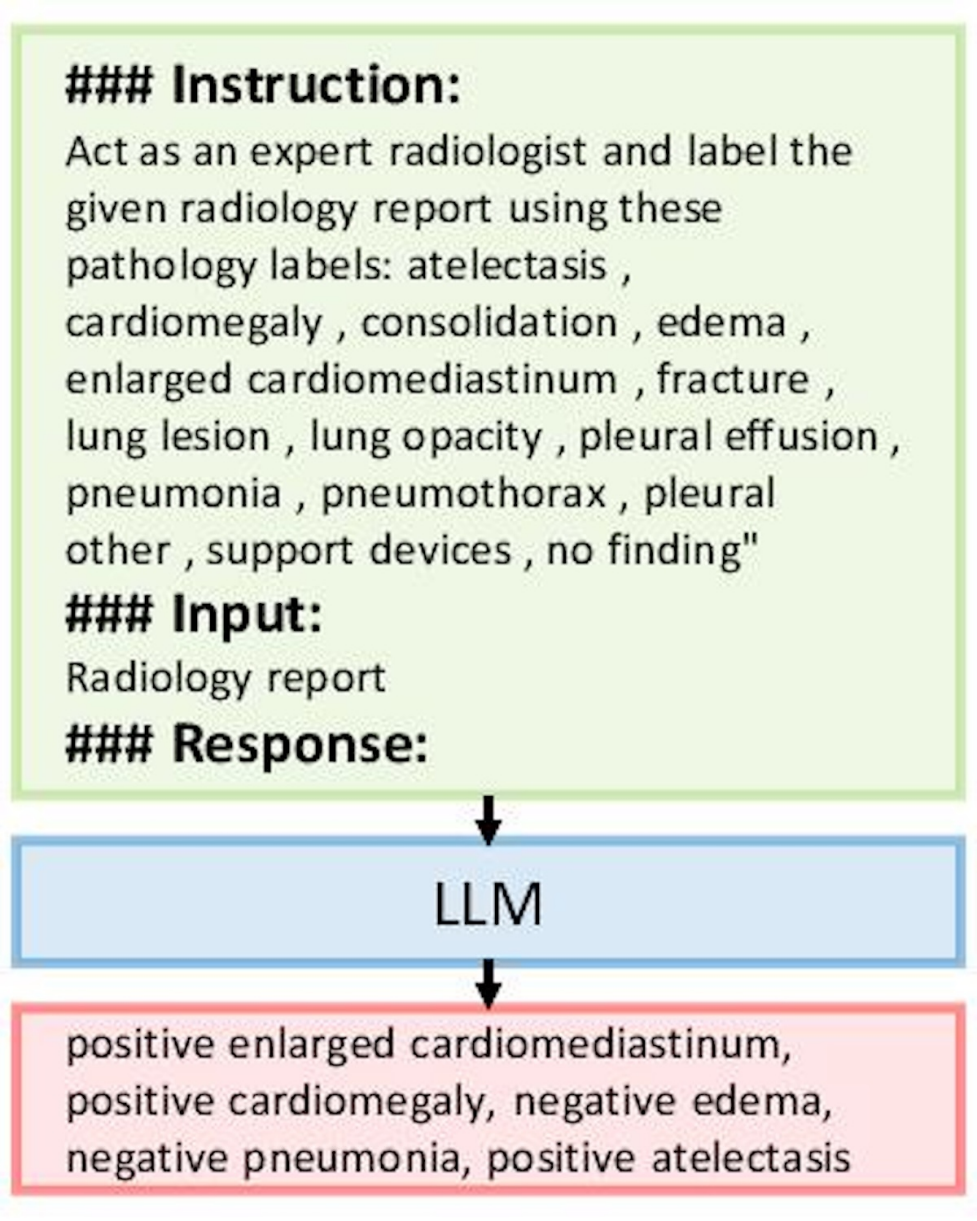
Instruction fine-tuning process for a large language model to label radiology reports. The dataset includes prompts structured to guide the large language model, input radiology reports, and corresponding pathology labels annotated with certainty levels. This method was applied to the MIMIC Chest X-ray dataset, a large-scale de-identified chest x-ray dataset containing radiologist-labeled reports. LLM: large language model

The structure of the instruction dataset is designed to facilitate clear guidance for the LLM. As illustrated in Figure 1, each data instance consists of an instruction in the form of a prompt to the LLM, an input value (the radiology report), and output values (the pathology labels along with their certainty levels). This structured approach helps the LLM understand both the format and task requirements, allowing it to generate accurate labels from free-text radiology reports.

To prepare the data for model processing, each instance in the instruction dataset is tokenized using the LLM’s tokenizer, converting text into a sequence of tokens suitable for input. The tokenized data is then fed into the LLM, which generates predictions based on its learned representations. Following this, we calculate the cross-entropy loss, which measures the difference between the model’s predicted outputs and the ground truth labels. This loss value indicates how closely the model’s predictions align with the actual labels. By back-propagating the loss, we adjust the LLM’s weights which gradually enhances its accuracy and reliability in producing pathology labels with certainty levels.

Through this fine-tuning process, our LLM becomes adept at associating the textual features of radiology reports with relevant pathology labels, allowing it to accurately and efficiently label clinical data. This approach ensures that the model is optimized specifically for radiology report labeling, enabling it to perform well even on complex clinical information.

### Training Details

We trained our LLM, which has a total of 463,987,712 parameters, using an instruction dataset. During the training process, we maintained a batch size of 2, which allows for effective gradient estimation while minimizing memory usage. This choice of batch size is particularly beneficial given the substantial size of our model and the complexity of the task. The training was conducted over 5 epochs, providing sufficient iterations for the model to learn the intricate relationships between the radiology reports and their corresponding pathology labels.

To optimize the training process, we used the ADAM optimizer [30] which facilitated faster convergence and improved performance. In addition, we implemented a gradient accumulation strategy with a value of 2. This approach effectively simulates a larger batch size by accumulating gradients over two iterations before performing a weight update, allowing us to maximize the use of available GPU memory while still benefiting from the stability of a larger batch size.

The learning rate was set at 1 × 10^−4^, a value that strikes a balance between training speed and stability. An appropriate learning rate is crucial in preventing the model from oscillating around the optimal solution, ensuring gradual and consistent improvements in performance. For the instruction tuning of our LLM, we used the SFTTrainer from the TRL library [31]. This specialized trainer is designed for supervised fine-tuning, providing an efficient and effective framework for adapting pretrained models to specific tasks. It offers a range of features that streamline the training process, including automated handling of training loops, logging, and monitoring of performance metrics. We leveraged 4 NVIDIA RTX A6000 GPUs. This powerful hardware setup enables parallel processing, significantly reducing training time while accommodating the memory requirements of our large model.

### Ablation Studies

#### Medical Contrastive Language-Image Pretraining Based Similar Reports Retrieval

To evaluate the effectiveness of our LLM based solution in its longer context we augment the LLM with similar retrieved reports which have been retrieved based on the cosine similarity, Medical Contrastive Language-Image Pretraining (MedCLIP) [32] is a Contrastive Language-Image Pretraining (CLIP)-based [33] model which is trained on medical images. It is trained in a constrastive manner and based on the cosine similarity between radiology images and texts it placed them closer or farther in its projection space. This helps in tasks such as image-image text-text or image-text and text-image based retrieval. To augment the input radiology report to the LLM, CheXbert, and CheXpert alike, we retrieve similar radiology reports from a datastore similar to [34]. MedCLIP has a limitation of 77 tokens as its context length and therefore text-text based retrieval is not possible. To overcome this, we use the radiology image associated with the radiology report of 687 radiologist annotated reports from MIMIC-CXR dataset and retrieve similar radiology images from the train set of MIMIC-CXR, this avoids any data leakage. Then the radiology reports corresponding to these top-k similar retrieved images is taken as the retrieved similar reports which are augmented to the test reports to increase the length of radiological text and test the abilities of labeling methods when longer context input is given. Retrieval is based on MedCLIP representations of input images and reports in the datastore, the datastore is precomputed offline and indexed with FAISS [35] for efficient nearest neighbor searching.

However, the performance of LLM dropped because the retrieved reports are not representative of the accurate information which can be used. Even though our model was able to process the additional information provided, it was unable to perform better because of the quality of those retrieved reports. In future with the advent of chat-bot style LLM solutions, longer bio-medical text in the form of patient-doctor conversation will be available and can be provided to our model as conversation history and this is where our model can be helpful.

### Ethical Considerations

To fully use the capabilities of our pretrained LLM, we fine-tune it using a specialized instruction dataset.

#### Human Subject Research Ethics Review

This study did not involve direct human subject research. Instead, it used the MIMIC-CXR dataset, a publicly available, large-scale radiology dataset. The creation and distribution of the dataset adhere to ethical standards, with data anonymized to protect patient privacy. Therefore, specific ethics review and approval for this secondary analysis were not required.

#### Informed Consent

The MIMIC-CXR dataset is derived from hospital records collected during routine clinical care. Informed consent for the use of this data was waived by the Institutional Review Board (IRB) of the Beth Israel Deaconess Medical Center (BIDMC), as the dataset underwent rigorous deidentification processes to ensure compliance with Health Insurance Portability and Accountability Act standards.

#### Privacy and Confidentiality

The dataset has been thoroughly deidentified to ensure anonymity. This includes removing all protected health information from radiology reports, applying optical character recognition and masking techniques to redact protected health information from images, and assigning random identifiers to patients, studies, and images. This guarantees that patient confidentiality is maintained in all analyses.

#### Compensation

No compensation was involved in the collection or use of the MIMIC-CXR dataset, as it is a retrospective collection of clinical records for research purposes.

#### Identification of Participants

There is no risk of identification of individual participants, as all data in the MIMIC-CXR dataset is fully anonymized. The deidentification process for radiology reports, images, and associated metadata adheres to strict privacy protocols to ensure that no identifying information is present in the dataset or the results of this study

### Evaluation

In this study, we evaluate the performance of our model, CheXbert and the CheXpert labeler, across a suite of retrieval tasks designed to assess their capabilities in clinical information extraction. Specifically, we focus on 3 main retrieval tasks, namely positive extraction, negative extraction, and uncertainty extraction. For each task, we designate the relevant class as the “positive” class for classification purposes, meaning, for example, that the “negative” class is treated as the positive class in the negative extraction task, while other classes (such as positive or uncertain) are treated as negatives. This approach allows us to directly measure the model’s ability to distinguish between the specified class and all others, thereby assessing its precision and recall within clinically relevant categories.

We compute the weighted average of the *F*1 scores for each of the 14 clinical findings or observations present in the CheXpert dataset across these tasks. *F*1 score is a harmonic mean of precision and recall which provides a balanced measure of a model’s classification performance, particularly in settings where data is imbalanced. We calculate a weighted average of the *F*1 scores for each of the 14 observations across these tasks. By this we can mitigate the impact of class imbalance and obtain an *F*1 metric that accurately reflects the model’s performance across both common and rare observations in the dataset.

This weighted metric, referred to as weighted-*F*1, is denoted simply as *F*1 in our results. Finally, we calculate and report the average *F*1 score across all 14 clinical observations, offering insight into the model’s general extraction capability. This average *F*1 score serves as a key performance metric, allowing for a direct comparison between CheXbert and the CheXpert labeler on clinical information extraction tasks relevant to medical imaging applications in radiology .

## Results

Table 1 and Table 2 present a quantitative assessment of our model’s performance relative to prvious BERT-based approaches for the task of radiology report labeling. Table 1 reports the *F*1 scores obtained by our model on the MIMIC-CXR dataset, specifically on a radiologist-labeled test set comprising 687 radiology reports. These scores encompass all certainty levels, positive, negative, and uncertain, across each of the 14 pathology categories. The results indicate that our model demonstrates superior performance over previous methods, particularly in the Enlarged Cardiomediastinum and Support Devices categories, while maintaining competitive F1 scores across the other pathology labels.

**Table 1.** *F*_1_ scores for predictions made by the proposed large language model-based model (ours) compared with CheXbert and CheXpert for all certainty levels (positive, negative, and uncertain). The evaluation was conducted on the MIMIC-CXR test set of 687 radiologist-labeled chest X-ray reports, covering 14 thoracic pathologies.

Pathologies	Ours, *F*_1_ score	CheXbert, *F*_1_ score	CheXpert, *F*_1_ score
Enlarged cardiomediastinum	0.9022	0.8753	0.8644
Cardiomegaly	0.8555	0.8604	0.8143
Lung Opacity	0.8653	0.8820	0.8459
Lung lesion	0.9612	0.9627	0.9543
Edema	0.9105	0.9191	0.9064
Consolidation	0.9288	0.9385	0.9215
Pneumonia	0.8784	0.8853	0.8474
Atelectasis	0.8613	0.8656	0.8576
Pneumothorax	0.9619	0.9780	0.9572
Pleural effusion	0.8510	0.8649	0.8475
Pleural other	0.9627	0.9623	0.9629
Fracture	0.9734	0.9758	0.9702
Support devices	*0.8607*	0.8402	0.8043
No finding	0.8470	0.8557	0.8557
Average	0.9014	0.9047	0.8864

**Table 2.** *F*_1_ scores for predictions made by the proposed large language model-based model (ours) compared with CheXbert and CheXpert for all certainty levels (positive, negative, and uncertain). The evaluation was conducted on the MIMIC-CXR test set of 687 radiologist-labeled chest X-ray reports, covering 14 thoracic pathologies.

Pathologies	Ours, *F*_1_ score	CheXbert, *F*_1_ score	CheXpert, *F*_1_ score
Enlarged cardiomediastinum	0.8907	0.8641	0.8447
Cardiomegaly	0.8233	0.8113	0.7650
Lung Opacity	0.8008	0.8230	0.7770
Lung lesion	0.9490	0.9507	0.9415
Edema	0.8850	0.8934	0.8805
Consolidation	0.9104	0.9222	0.9101
Pneumonia	0.8886	0.8907	0.8568
Atelectasis	0.8256	0.8316	0.8206
Pneumothorax	0.9505	0.9700	0.9470
Pleural effusion	0.7983	0.8143	0.7965
Pleural other	0.9448	0.9457	0.9466
Fracture	0.9626	0.9649	0.9581
Support devices	0.7911	0.7603	0.7069
No finding	0.7704	0.7835	0.7836
Average	0.8708	0.8733	0.8525

Table 2 further delineates our model’s performance by isolating only positive and negative certainty levels for each of the 14 pathology labels, thereby excluding uncertain cases to facilitate direct comparison. In this subset, our model yields notable improvements in *F*1 scores for Enlarged cardiomediastinum, cardiomegaly, and support devices.

The *P* values presented in Table 3 provide the results of statistical comparisons between the 3 models (ours, CheXbert, and CheXpert) based on their *F*1 scores. We performed both a paired *t* test and a Wilcoxon test to assess the statistical significance of the differences between the models. The paired *t* test *P* value for the comparison between ours and CheXbert is .35, which indicates that there is no statistically significant difference between the 2 models (*P*>.05). However, the comparison between ours and CheXpert yields a paired *t* test *P* value of .01, suggesting that ours performs significantly better than CheXpert at the 5% significance level (*P*<.05). Similarly, the Wilcoxon test confirms this finding with a *P* value of .005. The comparison between CheXbert and CheXpert shows highly significant differences with both a paired *t* test *P* value of .0005 and a Wilcoxon test *P* value of .002, indicating that these 2 models also differ significantly in performance. Overall, the statistical tests reveal that while ours and CheXbert are statistically similar, ours outperforms CheXpert, and there is a significant performance difference between CheXbert and CheXpert.

**Table 3.** *P* values from statistical tests (paired *t* test and Wilcoxon test) comparing the performance of the proposed LLM-based model (ours) with CheXbert and CheXpert across 14 thoracic pathologies. The evaluation was conducted on the MIMIC-CXR test set of 687 radiologist-labeled chest X-ray reports, assessing the significance of the differences in *F*_1_ scores for all certainty levels.

Comparison	Paired *t* test *P* value	Wilcoxon test *P* value
Ours versus CheXbert	.35	.14
Ours versus CheXpert	.01	.005
Chexbert versus CheXpert	.0005	.002

Compared with the rule-based CheXpert labeler [8], our model achieves substantial performance gains across all pathology labels, marking a significant advancement over both traditional rule-based systems and BERT-based models. These results demonstrate the ability of our large language model (LLM)-based approach in handling the task of radiology report labeling with greater precision with large context length as compared with the small context length of BERT based models.

**Figure 2.**
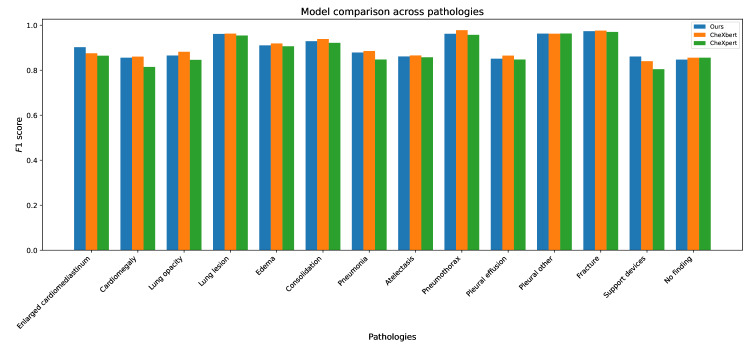
Performance comparison of the proposed large language model-based model (ours), CheXbert, and CheXpert across 14 thoracic pathologies. The *F*_1_ scores for each pathology label were calculated using the MIMIC-CXR test set, comprising 687 radiologist-labeled reports. While all models show similar performance, the proposed model demonstrates competitive or superior results in cases such as “support devices” and “no finding.”

Figure 2 presents the performance of the proposed model (ours) compared with the baseline models (CheXbert and CheXpert) across 14 chest pathologies, using *F*1 scores as the evaluation metric. The grouped bar chart enables a direct comparison, where each group corresponds to a specific pathology, and individual bars represent the scores for each model. The results reveal that all three models achieve consistently high *F*1 scores across most pathologies, indicating robust performance. While the differences are marginal, notable trends can be observed, the proposed model demonstrates an advantage in pathologies such as support devices and enlarged cardiomediastinum, whereas the baseline models perform slightly better in cases like consolidation and pneumothorax. This chart highlights the subtle strengths and weaknesses of each model, providing a clear visual overview of their performance.

**Figure 3.**
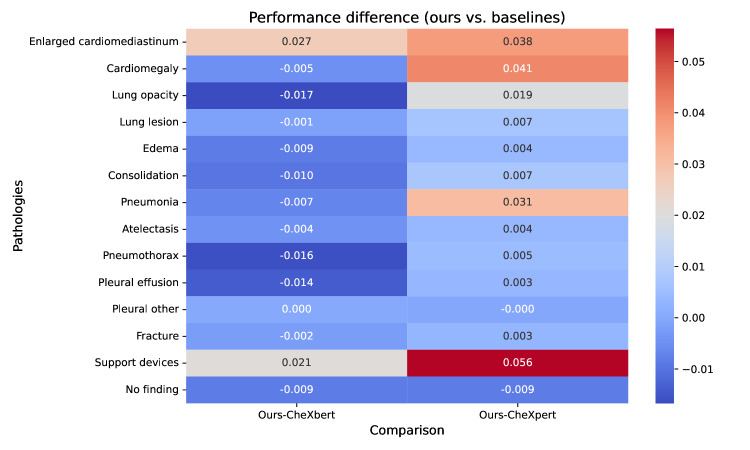
Heatmap showing the absolute differences in *F*_1_ scores between the proposed arge language model-based model (ours) and the baseline models (CheXbert and CheXpert) across 14 thoracic pathologies. The analysis is based on predictions for the MIMIC-CXR test set of 687 radiologist-labeled reports. The heatmap highlights areas where ours excels, such as “support devices,” and where baseline models perform better, such as “consolidation.”

Figure 3 further explores the differences by visualizing the absolute performance gaps between the proposed model (ours) and the 2 baseline models using a heatmap. Rows correspond to the pathologies, and columns display the comparisons of ours against CheXbert and CheXpert. The heatmap emphasizes that the differences are minor, with most pathologies showing a difference of less than 0.02 in *F*1 scores. However, it also highlights key instances where ours performs notably better, such as in support devices and enlarged cardiomediastinum, while the baselines excel slightly in consolidation and pneumothorax. By focusing on performance differences, the heatmap provides a nuanced perspective, making it easy to identify pathologies where the models vary.

## Discussion

### Limitations

Currently, our work is limited to 14 pathology labels prevalent in radiology. However, our method can be extended to other medical fields and can accommodate many more pathology labels and can provide a reliable labeling solution. The lack of long form radiology text limits the demonstration of the longer contextual abilities of our model. In future with the advent of long form bio-medical text in the form of patient-doctor conversations, our model can provide better solutions as opposed to previous models. Furthermore, there is a need of more publicly available radiologist annotated datasets for additional evaluation of trained models. Furthermore, like other LLM-based approaches, our model is susceptible to biases present in the training data, which may inadvertently propagate into its predictions. This is particularly important in the medical domain, where biases can have significant ethical and clinical implications. Additional mechanisms for bias detection and mitigation are necessary to ensure fair and equitable outcomes across diverse patient populations.

The computational demands of LLMs are another limitation, as training and inference require substantial computational resources. This may limit the accessibility and scalability of the approach, especially in resource-constrained clinical settings. Exploring techniques such as model distillation or low-rank adaptation could help reduce these requirements while maintaining performance.

Interpretability remains a challenge with LLMs due to their complex architecture. The lack of transparency in how decisions are made can hinder trust and adoption in clinical workflows, where clear justification of predictions is often required. Future work should explore ways to improve the explainability of LLM predictions, such as attention visualization or feature attribution methods tailored to medical tasks.

While our method demonstrates promising results in radiology, further validation is needed in real-world clinical settings and across diverse medical contexts. The scalability of our approach to other modalities, such as MRI or CT reports, and its adaptability to different healthcare systems must also be explored to ensure generalizability.

### Principal Findings and Conclusions

In our work, we propose a novel solution using LLMs for the challenging task of radiology report labeling. Our approach leverages the advanced capabilities of LLMs to accurately interpret and label various pathologies within radiology reports, which are crucial for supporting clinical decision-making and improving patient care. We demonstrate that LLMs achieve superior performance over previous BERT-based models, especially in accurately identifying specific pathology labels. This is a significant advancement, as certain pathologies that were previously challenging to label accurately can now be identified with greater precision using our LLM-based approach. In addition, our model achieves competitive scores across a broad range of pathology labels, confirming its robustness and versatility in handling complex medical language.

One of the key improvements introduced by our model is its ability to process longer radiology reports, overcoming the context length limitations often encountered in previous models. This capability is essential in clinical settings where comprehensive radiology reports often contain detailed descriptions spanning multiple paragraphs, which traditional models struggle to handle effectively. By accommodating extended context, our model ensures that no critical information is overlooked, thereby enhancing labeling accuracy and reliability in real-world applications.

Furthermore, the instruction-tuning methodology allows our approach to be extended to other biomedical text labeling tasks beyond radiology. This generalizability of our method makes it highly applicable across a wide range of biomedical domains, opening doors for enhanced automation in various clinical documentation and reporting tasks.

As LLM-based solutions are increasingly adopted in clinical settings, our model is designed to integrate seamlessly into existing workflows, promoting efficiency, and facilitating clinical automation. The potential for real-time, accurate labeling provided by our model not only reduces the manual workload on health care professionals but also contributes to more timely and precise diagnostic insights, ultimately benefiting patient outcomes.

Future research should explore integrating LLMs with other medical technologies, such as multimodal imaging systems that combine radiology reports with visual data from x-rays, CT scans, or magnetic resonance images. This integration could enable a more comprehensive analysis and further enhance the clinical utility of LLM-based models. In addition, extending the application of LLMs to different medical imaging modalities and nonradiology domains, such as pathology or cardiology, presents a promising avenue for expanding their impact across health care.

Developing scalable and efficient LLM solutions tailored to specific clinical needs, including resource-constrained environments, is another critical area for further exploration. This includes investigating lightweight alternatives or model compression techniques to reduce computational demands while maintaining performance.

Finally, the clinical adoption of LLMs will require continued efforts in improving model interpretability and building trust among health care professionals. Future work should focus on developing user-friendly interfaces and explainability mechanisms to facilitate the seamless integration of LLMs into routine clinical workflows. By addressing these challenges, LLM-based models like ours have the potential to revolutionize clinical practice, enabling more accurate, efficient, and scalable solutions for medical documentation, decision-making, and patient care.

## Abbreviations

BERT: Bidirectional Encoder Representations from Transformers
CLIP: Contrastive Language-Image Pretraining
GPT: generative pretrained transformer
LLM: large language model
MedCLIP: Medical Contrastive Language-Image Pretraining
NRF: National Research Foundation of Korea
